# The trajectory of intrahelical lesion recognition and extrusion by the human 8-oxoguanine DNA glycosylase

**DOI:** 10.1038/s41467-020-18290-2

**Published:** 2020-09-07

**Authors:** Uddhav K. Shigdel, Victor Ovchinnikov, Seung-Joo Lee, Jenny A. Shih, Martin Karplus, Kwangho Nam, Gregory L. Verdine

**Affiliations:** 1 Department of Stem Cell and Regenerative Biology, Harvard University, Cambridge, MA 02138 USA; 2 Department of Chemistry and Chemical Biology, Harvard University, Cambridge, MA 02138 USA; 3 Laboratoire de Chime Biophysique, Institut de Science et d’Ingénierie Supramoléculaires, Université de Strasbourg, 67000 Strasbourg, France; 4 Department of Chemistry and Biochemistry, University of Texas at Arlington, Arlington, TX 76019-0065 USA; 5 Department of Chemistry, Umeå University, Umeå, SE 901 87 Sweden; 6 Present Address: LifeMine Therapeutics, 430 East 29th Street, Suite 830, New York, NY 10016 USA; 7 Present Address: Beam Therapeutics, 26 Landsdowne Street, 2nd Floor, Cambridge, MA 02139 USA; 8 Present Address: Beth Israel Deaconess Medical Center, 330 Brookline Avenue, Boston, MA 02215 USA

**Keywords:** Structural biology, Computational biophysics, Molecular biophysics

## Abstract

Efficient search for DNA damage embedded in vast expanses of the DNA genome presents one of the greatest challenges to DNA repair enzymes. We report here crystal structures of human 8-oxoguanine (oxoG) DNA glycosylase, hOGG1, that interact with the DNA containing the damaged base oxoG and the normal base G while they are nested in the DNA helical stack. The structures reveal that hOGG1 engages the DNA using different protein-DNA contacts from those observed in the previously determined lesion recognition complex and other hOGG1-DNA complexes. By applying molecular dynamics simulations, we have determined the pathways taken by the lesion and normal bases when extruded from the DNA helix and their associated free energy profiles. These results reveal how the human oxoG DNA glycosylase hOGG1 locates the lesions inside the DNA helix and facilitates their extrusion for repair.

## Abstract (web summary)

DNA glycosylases are lesion-specific enzymes that recognize specific nucleobase damages and catalyze their excision through cleavage of the glycosidic bond. Here, the authors present the crystal structures of human 8-oxoguanine (oxoG) DNA glycosylase bound to undamaged DNA and to DNA containing an intrahelical oxoG lesion and further analyse these structures with molecular dynamics simulations, which allows them to characterise the base-extrusion pathways.

## Introduction

The chemical instability of the genome toward attack by a variety of reactive species with which it comes into routine contact has given rise to dedicated surveillance and maintenance systems that seek out DNA damage and repair it. Most forms of damage to the heterocyclic nucleobases of DNA are repaired by mechanistically related pathways collectively referred to as the base-excision repair (BER) mechanism. Both the recognition of damaged sites and initiation of repair by BER are performed by DNA glycosylases, lesion-specific enzymes that recognize specific nucleobase damages in the genome and catalyze their excision through cleavage of the glycosidic bond^1–4^.

**Fig. 1:**
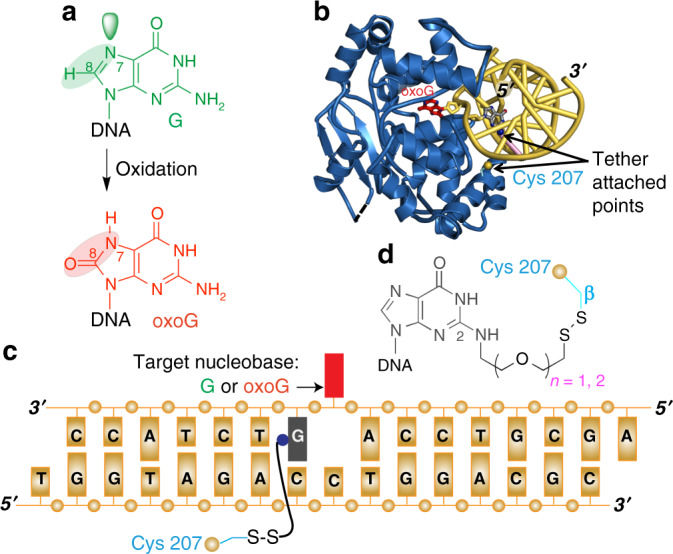
Generation of 8-oxoguanine and experimental strategy used to capture hOGG1 interrogating DNA. **a** Structural comparison of G versus oxoG. **b** Structure of un-crosslinked lesion-recognition complex, LRC^16^, of hOGG1 (cyan) bound to oxoG containing DNA (red). Blue sphere on the guanine (gray) represents the point of thiol-tether attachment and the yellow sphere represents Cys residue used for crosslinking. **c** Schematic DNA sequence diagram illustrating the crosslinking site in relation to the target nucleobase. **d** Attachment of the thiol-tether at the N2 position of guanine, with the tether protruding into the minor groove.

DNA glycosylases carry out the formidable task of locating, on average, one aberrant base embedded among ~10^6^–10^7^ normal bases^5^. Many of these lesions differ from their undamaged counterparts by at most a few atoms and cause no frank distortion or little energetic destabilization of the DNA duplex^6–9^. Among them, 8-oxoguanine (oxoG, ^O^G) presents the greatest challenge to the DNA glycosylases responsible for its repair, due to its unique combination of structural innocuousness, extrordinary mutagenicity, and chronic low-level production. Arising through the attack of the reactive by-products of aerobic respiration on guanine (G), oxoG differs from G by only two “atoms”, =O versus –H at C8, and lone-pair versus –H at N7 (Fig. 1a). Also, oxoG is highly mutagenic and mis-pairs with adenine at a greater than 90% frequency during processive DNA replication^10^. For these reasons, oxoG is believed to be responsible for the majority of G:C to T:A transversion mutations, which are the second most common type of spontaneous genetic change in humans^11^. The G:C to T:A transversion mutation has also been found in codon 12 of the highly oncogenic protein K-ras, which resulted in the formation of lung tumors in mice deficient in the oxidative DNA repair genes, *myh* and *ogg*^12^. Transversion mutations by oxoG play the important role of creating genetic diversity in humans as well as in other organisms. In particular, a high density of oxoG was discovered in regions with a high frequency of recombination and single nucleotide polymorphisms (SNPs)^13^. An increased incidence of G−to−T mutations was also found in offsprings of a MTH1/OGG1/MUTYH triple knockout mouse suggesting that oxoG is responsible for spontaneous and inheritable mutations of the germ lineage cells^14^.

Extensive studies of DNA glycosylases have revealed the molecular mechanism through which they specifically recognize rare and pernicious adducts, such as oxoG and 3-methyadenine, and initiate their catalytic removal^15–17^. High-resolution crystal structures of the aborted catalytic complexes of DNA glycosylases bound to cognate lesions have revealed that all DNA glycosylases extrude their substrate nucleoside completely from the DNA helix and insert it into an extrahelical lesion-recognition pocket of the enzyme (as in Fig. 1b)^15,16,18^. Such a complex is referred to as a lesion recognition complex (LRC)^15,16^, and the structures of extrahelical lesion bound complexes are indispensable to understand lesion recognition mechanism. However, while searching for lesions, the enzyme translocates along DNA at nearly the limit of 1-dimensional diffusion, eliminating the possibility that every DNA base is extruded into the enzyme’s active site for lesion inspection^19^. This suggests that DNA glycosylases must recognize the lesion while it is fully nested in the DNA duplex helical stack. The nature of this early intrahelical lesion-encounter event remains poorly understood.

To understand the early events by which DNA glycosylases locate the lesion and initiate extrusion, we have used synthetic crystallography to capture the otherwise fleeting intermediates that arise as a DNA glycosylase interrogates the genome for the presence of lesions. At the heart of the synthetic crystallography is a technique developed in our laboratory called intermolecular disulfide crosslinking (DXL) that restricts the roaming range of the protein on DNA, thereby limiting the population size of the non-specific DNA–protein complex ensemble. We and others have shown that this strategy is effective in producing homogenous complexes amenable to crystallization and structural elucidation^15,20–27^.

Using DXL technology, we have previously found an X-ray structure of bacterial 8-oxoguanine DNA glycosylase, MutM, interrogating a fully intrahelical lesion oxoG^22^. This structure, with biophysical and computational studies on the MutM recognition of oxoG, has led to the most comprehensive understanding of the lesion-search and repair mechanisms determined for any DNA glycosylase^22,28–30^. The question addressed here is whether the 8-oxoguanine DNA glycosylase responsible for oxoG recognition and repair in humans, namely hOGG1, interrogates DNA in the same way as the functionally equivalent bacterial enzyme MutM, despite the lack of an overall structural and sequence similarity between these two oxoG repair enzymes. Using the enhanced DXL technology developed in this study, we report the crystal structures of hOGG1 with a fully intrahelical target G•C base-pair and a sequence-matched complex bearing a fully intrahelical target oxoG•C base-pair. By adopting the notation used for MutM, these two complexes are referred to as the interrogating complex (IC) and the encounter complex (EC), respectively.

## Results and discussion

### Entrapment of hOGG1 complex with intrahelical DNA

Numerous structures of hOGG1 complexed with DNA have been reported, but all represent states with the target nucleotide in the course of being extruded from the DNA helix^16,23,31–33^. To entrap the complex of hOGG1 with intrahelical G or oxoG, we identified several potential sites for DXL in the protein–DNA interface based on the native lesion-recognition complex (LRC) of hOGG1^16^ and prepared the corresponding mutant proteins, containing a single cysteine at the relevant positions. Given that, a panel of oligonucleotides shown in Fig. 1c containing a thiol-tether attached to the major grove, the minor grove, or the DNA backbone were screened for DXL. In this study, we developed 5 and 8 atom long new tethers, –CH_2_CH_2_OCH_2_CH_2_–, –CH_2_CH_2_OCH_2_CH_2_ OCH_2_CH_2_–, hereafter referred as X_5_ and X_8_ (see Supplementary Fig. 1 for synthesis) respectively, that do not suffer from the insolubility problems that hampered past attempts to incorporate long hydrocarbon linkers. Tethers were systematically varied in length and composition from C_2_ (–CH_2_CH_2_–) to C_3_ (–CH_2_CH_2_CH_2_–), C_4_ (–CH_2_CH_2_CH_2_CH_2_–), X_5_ (–CH_2_CH_2_OCH_2_CH_2_–), and X_8_ (–CH_2_CH_2_OCH_2_CH_2_OCH_2_CH_2_–). On the basis of this screen, we have selected the Y207C variant, because it crosslinks efficiently but is substantially distant from the active site of the enzyme to participate in the catalysis and does not interact with DNA in native LRC, and crosslinked it through the X_8_ linker to the minor groove N2-exocyclic amine of 3′ guanine of the target nucleobase (Fig. 1b–d). The long X_8_ linker (~17 Å long) has the flexibility necessary for the capture of the hOGG1 complex with intrahelical DNA. The complex produced a crystal structure at 2.35 Å (Supplementary Table 1) resolution with an intrahelical target G•C base-pair, hereafter referred to as the interrogation complex (IC).

**Fig. 2:**
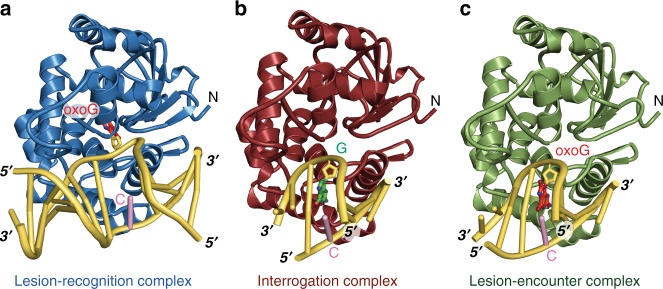
Interaction of human 8-oxoguanine DNA glycosylase I (hOGG1) with DNA. **a** Overall structure of a crosslinked lesion-recognition complex, xLRC, with an extrahelical oxoG bound in the enzyme active site. **b** Structure of the sequence-matched interrogation complex, IC, with a fully intrahelical target G•C base-pair. **c** Structure of the sequence-matched encounter complex, EC, with a fully intrahelical target oxoG•C base-pair.

To entrap the structure of hOGG1 encountering the intrahelical oxoG•C lesion, a point mutation, C253W, was introduced in the active site to sterically block the entrance of oxoG to the active site^34^, since a similar strategy had proved successful in related studies with MutY. With this and Y207C variant tethered to X8 linker, the sequence-matched structure of hOGG1 encountering an intrahelical oxoG•C lesion, referred to as the EC, was obtained and refined to 2.38 Å resolution (Supplementary Table 1). In both IC and EC structures, the target G•C and oxoG•C base-pairs are unambiguously intrahelical (Fig. 2b, c and Supplementary Fig. 2a, b), with a root-mean-square deviation (RMSD) of 0.244 Å between the two structures, in which the RMSD superposition was performed for all atoms. These structures reveal the nature of early events in DNA inspection of hOGG1.

In parallel, we solved the structure of the control complex (2.37 Å resolution, Supplementary Table 1) to show that DXL between Y207C and the X8 linker does not interfere with oxoG extrusion and recognition by hOGG1. The structure of this complex, herein xLRC (with catalytically inactive K249Q), reveals that the active site as well as the overall structure is identical within an RMSD of 0.201 Å for Cα atoms (a total of 2058 atoms) to those of the native LRC^16^. In the xLRC structure the oxoG is extrahelical and deeply inserted into the enzyme’s lesion recognition pocket as observed in the native LRC complex^16^ (Fig. 2a and Supplementary Fig. 3a), confirming that the introduced mutations and crosslinking through the X8 linker do not interfere with the extrusion of oxoG into the active site of hOGG1.

### Unique Interactions of hOGG1 with DNA

Several features of the IC and EC structures support the conclusion that the two structures represent the state of the enzyme at its initial encounter with the DNA. They are characterized below in terms of three key elements, which are different from the native LRC: (1) unique conformation of the DNA backbone, (2) rearrangement of the active site, and (3) different interaction with the C opposite oxoG (hereafter referred to as the estranged C).

**Fig. 3:**
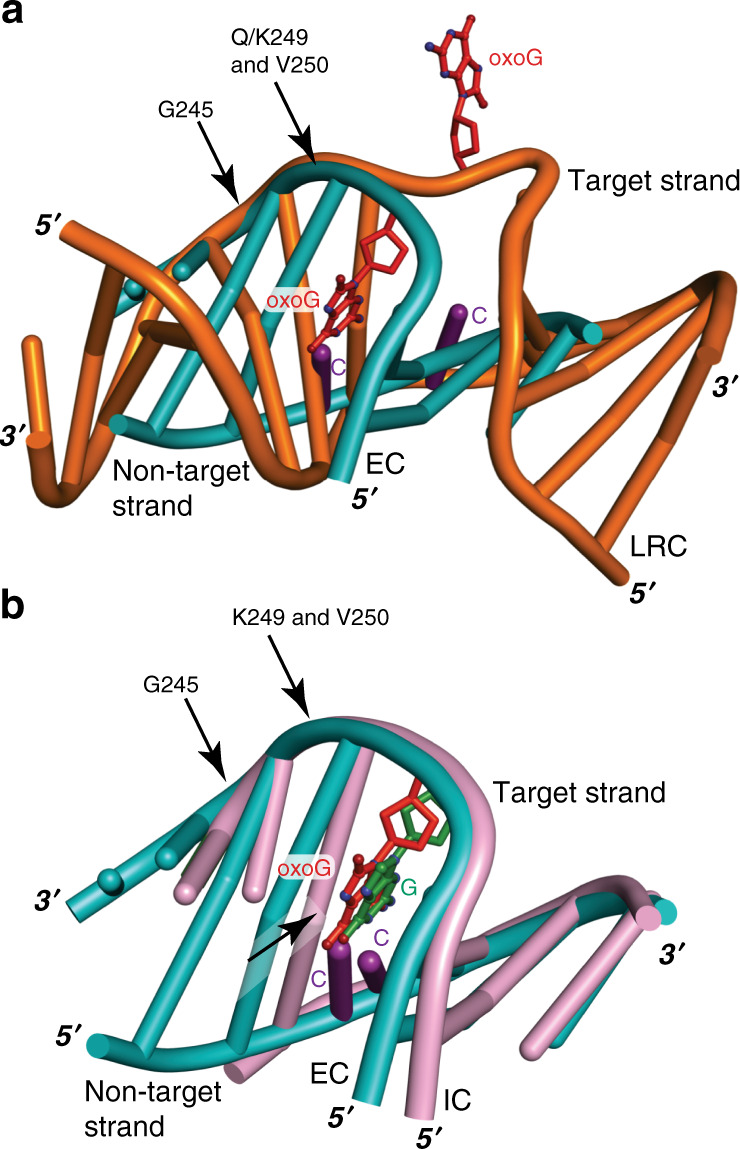
Comparison of the DNA conformations between the sequence-matched xLRC, EC, and IC. Signature helix-hairpin-helix motif residues G245, Q/K249, and V250 that interact with DNA through backbone amide, common to all hOGG1–DNA structures solved to date, are denoted by an arrow. DNA base-pairs are shown as orange ladders in xLRC, teal ladders in EC, and pink ladders in IC. The target base is shown as sticks (red: oxoG; green: G) and estranged cytosine is labeled as purple. **a** Superposition of xLRC with EC. The DNA trajectory of the 5′ end of the target strand and the entire non-target strand is strikingly different between EC and xLRC structures. **b** Superposition of EC with IC. In both structures, the target strand remains in the same position with respect to hOGG1, but the non-target DNA strand of the IC structure has translocated by half a nucleotide step toward the 3′ end of the non-target strand, marked with an arrow.

First, the DNA conformation of the IC and EC structures differs substantially from that of LRC. The least-squares superposition of the IC and EC structures with LRC, using only the protein component in the superposition, clearly shows a well-defined anchor point on the 3′ side of the target strand (Fig. 3). The backbone of the target strand is held in place by the main-chain hydrogen bonds with G245, Q/K249 and V250 of the signature helix-hairpin-helix motif, which are found in all hOGG1–DNA structures solved to date^16,23,31–33^. On the other hand, the 5′ side of the target strand and the non-target strand of the IC and EC structures are noticeably different from those of LRC (Fig. 3).

Secondly, in the LRC, F319 and C253 interact with both π-faces of the extruded oxoG, which sandwich the base in the active site of hOGG1. The extruded oxoG is further stabilized by hydrogen bonding with the sidechain carbonyl of Q315 and also with the backbone carbonyl of G42 (Supplementary Fig. 3a). The interaction with G42 is specific to oxoG and contributes to the discrimination of oxoG in the active site^16,23^. These interactions are not possible in the IC and EC structures as there is no base extrusion. In addition, the αO-helix, which includes F319 and Q315 in the IC and EC structures, retracts away from that of LRC (Supplementary Fig. 3b, c), leaving the active site open for lesion binding. This active site rearrangement was observed previously in the exo-site structure of hOGG1^23^ (Supplementary Fig. 3d), suggesting that the rearrangement of the αO-helix relative to LRC takes place after or in concert with the insertion of the base into the active site^16,23^.

**Fig. 4:**
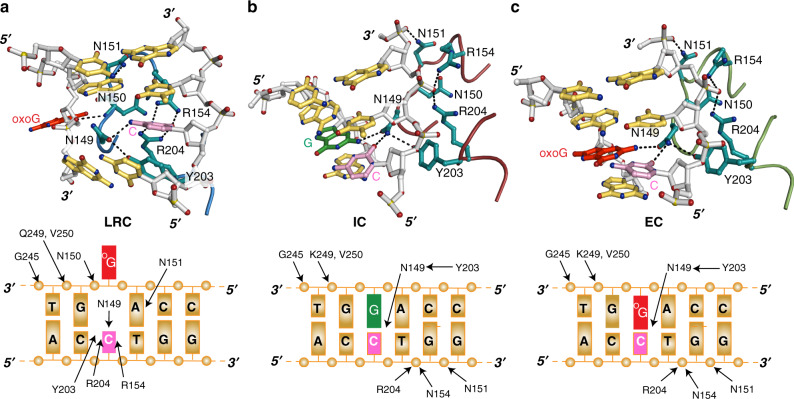
Close-up views of key interactions of hOGG1 with DNA. **a** Crosslinked LRC; **b** IC; and **c** EC. Dashed lines indicate hydrogen bonding interactions among DNA bases, backbone and residues in hOGG1. Color-coding is as in Fig. 2, except for the sidechains of key residues (N149, N150, N151, R154, Y203, and R204), which are shown in cyan. In each figure, the lower panel is a schematic DNA sequence diagram illustrating the residues that interact with DNA shown in the upper panel. The interactions are indicated with arrows.

Finally, hOGG1 in the IC and EC structures interacts differently with the estranged C (Fig. 4). In LRC^16^, Y203 wedges into the DNA helical stack on the 5′-side of the estranged C stabilizing a helical bend. N149 enters the space left vacant by the oxoG extrusion and hydrogen bonds with the Watson-Crick face of the estranged C. In addition, the estranged C is stabilized by R154 and R204 that form bidentate hydrogen bonds together with N149. On the target strand, the 3′ and 5′ phosphoryl groups of oxoG are anchored to hOGG1 by main chain hydrogen bonds with N150 of the conserved NNN motif (Fig. 4a). This mode of interaction was observed in all the hOGG1–DNA complex structures published to date, including the exo-site structure^23^.

In the IC and EC structures, due to the presence of oxoG/G in the helical stack, the residues mentioned above engage in a different mode of interactions (Fig. 4b, c). For example, R154 and R204 contact the non-target strand backbone, instead of interacting with the C opposite to oxoG (i.e., the estranged C in LRC). In addition, Y203 does not invade the helical stack and remains at the periphery of the minor-groove face of DNA. Similarly, N149 rests on the minor-groove face of the target G•C (in IC)/oxoG•C base-pair (in EC) and hydrogen bonds with both bases (Fig. 4b, c and Supplementary Fig. 4a–c). In these hydrogen bonds, the interaction with N2 of G/oxoG is specific to G and oxoG. Adenine (A) cannot interact with N149 in this orientation (Supplementary Fig. 5). For C and T, although their C2=O carbonyl can form a hydrogen bond with N149, its sidechain is too short to reach C and T on the target strand. OxoG can also adopt a syn conformation and mis-pair with adenine (Supplementary Fig. 5d). Although N149 could in principle interact with the C8=O in the syn conformation, similar to C and T, the side chain of N149 is too short to reach the C8=O group of oxoG (syn), so it is unlikely that N149 will make a productive engagement with the oxoG (syn):A pair. Biochemical data also support that hOGG1 is specific to oxoG(anti):C and does not catalyze oxoG (syn):A pair effeciently^35^.

### Intrahelical lesion recognition by hOGG1

Between IC and EC, while the target strand follows a similar backbone trajectory, the non-target strand of IC has translocated a half-nucleotide step toward its 3′ end relative to EC (Fig. 3b). Despite the conformational difference of DNA between the IC and EC structures, their DNA backbone structures around the target nucleotide are similar to each other (Supplementary Fig. 6a). The only notable difference is in the longer distance between C8 and C5′ of oxoG compared to the corresponding distance of IC (5.4 Å in EC vs 4.4 Å in IC).

The X-ray structures also reveal that, in EC, a water molecule bridges the oxoG to its 5′-backbone phosphate (Supplementary Fig. 6b) so as to attenuate the repulsion between them. Previously, it had been shown that the repulsion between C8=O of oxoG and its backbone phosphate plays a key role in the oxoG-specific intrahelical recognition by MutM^22^. The repulsion forces the oxoG ribose to adopt an alternative sugar pucker and/or a rotation of phosphodiester groups around oxoG. In the EC structure of hOGG1, the longer distance and the bridging water molecule between C8=0 of oxoG and the C5′ and 5′-backbone phosphate, respectively, could help bend the DNA at the target site, bringing these repulsive functional groups (i.e., oxoG and backbone phosphate) close to each other, thereby initiating base extrusion. Through this repulsion the enzyme discriminates oxoG and G, even at the initial encounter of the (intrahelical) lesion, prior to base extrusion and the conformational change to LRC.

MD simulations reveal an important consequence of the structural differences at the target site between EC and IC. As presented in Supplementary Fig. 7, in the presence of oxoG, hOGG1 establishes a stable contact with the target strand, while in the case of G it fluctuates back-and-forth. Consistent with this, the target strand with oxoG shows smaller root-mean-square fluctuation (RMSF) of atomic position than that with G (Supplementary Fig. 8). This suggests that the enzyme can quickly translocate to the next base pair in IC, while in the case of oxoG, it is locked at the target site.

### Lesion discrimination and base extrusion mechanism

**Fig. 5:**
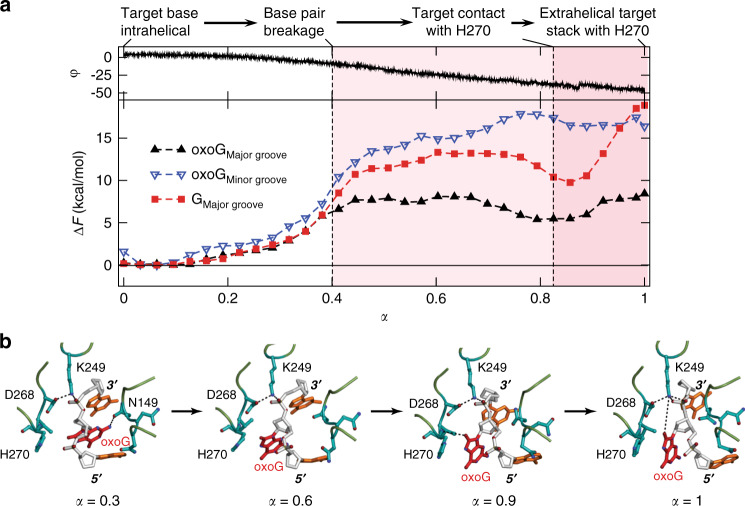
Free energy profiles determined for the oxoG and G extrusions. **a** Free energy profiles of base extrusion by hOGG1 described by a progress variable α (see Supplementary Fig. 9 for details) between the intrahelical IC (for G) or EC (for oxoG) state (α = 0) and the fully extrahelical state (α = 1). Important events along the major groove base extrusion path for oxoG are indicated together with the pseudo-rotation angle φ (Supplementary Fig. 10b) describing the target base extrusion. **b** Snapshots from the string method in collective variables (SMCV) simulations^36^, showing the interaction of oxoG with N149, K249, and H270. Key protein sidechains are shown in cyan, DNA phosphate and sugar backbones in gray, and DNA bases in orange, respectively.

In the IC and EC structures, hOGG1 does not form any direct interaction with the discriminatory major groove face of oxoG and G (Fig. 1a). This raises the question: by what mechanism does hOGG1 discriminate G and oxoG in the early phase of extrusion? To answer this question, we simulated the extrusion of G and oxoG out of the DNA helix into an extrahelical state and determined the associated free energy change by use of the string method^36^. The free energy profiles determined for the oxoG and G extrusions in this event are presented in Fig. 5a, in which the progression of the base extrusion is described by a normalized reaction coordinate α. The simulations show that the extrusion proceeds in three steps: (1) the target base is extruded out of a helical stack through a major groove of DNA, (2) the extruded base binds transiently at the exo-site, and (3) it then enters the active site of the enzyme with concomitant closure of the active site (see Supplementary Movie 1).

**Fig. 6:**
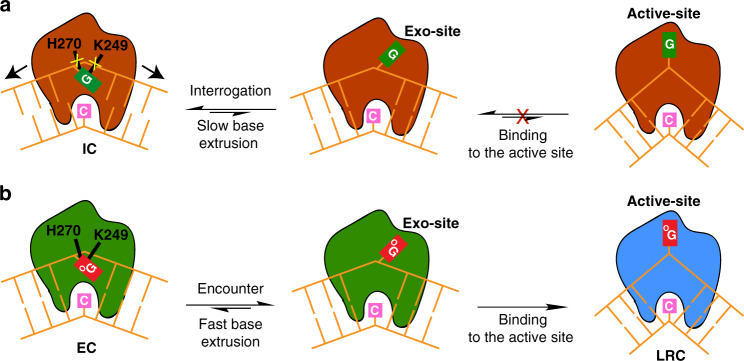
Discrimination of oxoG (^O^G) versus G. **a** H270 and K249 do not engage with G in IC, thus there is no or slow base extrusion, while the enzyme fluctuates back-and-forth along the target DNA strand. **b** H270 and K249 specifically recognize oxoG and facilitate the extrusion of oxoG. It then enters the active site of the enzyme to initiate its catalytic repair.

The MD simulation shows that G has a higher barrier than oxoG (13.3 kcal/mol for G versus 8.1 kcal/mol for oxoG; Fig. 5a). Moreover, the free energy of G continues to increase to 21.2 kcal/mol near the exo-site (Supplementary Fig. 9). Two protein residues, H270 and K249, stabilize the extruded oxoG through C8=O but not G (Fig. 5b); both residues are indispensable for the oxoG cleavage^37^. In particular, H270 forms a hydrogen bond with C8=O of the extruded oxoG between α = 0.5 and 0.9, through its backbone amine (Fig. 5b); Supplementary Fig. 9 shows the change of the distance between H270 and oxoG C8=O group along the entire base extrusion process. As the oxoG extrusion continues, the C8=O group begins to interact with the K249 sidechain. These interactions lead to a relatively flat free energy profile between α = 0.5 and 1.0 (Fig. 5a). Since these interactions are not possible for G, its free energy remains high, thus G return quickly to the intrahelical position. This difference suggests that hOGG1 kinetically discriminates the DNA lesion during its extrusion as shown in Fig. 6.

Despite the significant difference of the free energy barrier between oxoG and G, the free energy of the two systems increases very similarly at the beginning of the base extrusion between 0 < α < 0.4 (Fig. 5a). This is consistent with the fact that hOGG1 does not interact directly with the N7 and C8 of oxoG and G in the IC and EC structures, thus not discriminating between them in the early phase of base extrusion. Poor discrimination in the early phase of base extrusion was also proposed from the stopped-flow fluorescence measurements^38,39^. Nevertheless, compared with similar events in water^40,41^, the entire process is accelerated by DNA bending and the extent of protein–DNA contacts on the minor groove face of DNA. For example, hOGG1 uses a non-specific breakage of the target base-pair, assisted by N149. In addition, K249 contacts the 3′-side phosphate of oxoG/G at the beginning of the process (Figs. 5b, 6), thereby establishing a pivot for base extrusion. H270 is the first residue that specifically interacts with extruded oxoG, followed by K249 with C8=O of oxoG. This suggests that H270 and K249 function as a “cherry-picking” residue in hOGG1, with a similar role of R112 in MutM via significantly different mechanisms^22^. By contrast, in the case of G, its extrusion cannot be stabilized by the two residues and competes with the translocation of the enzyme along the DNA strand.

Figure 5 also suggests that hOGG1 extrudes the oxoG through the major groove, in accordance with the previously determined hOGG1/DNA complex structure^32^ with a barrier of 8.1 kcal/mol. The free energy profile for the minor-groove oxoG extrusion is also presented in Fig. 5a, and the free energy profiles along the entire base extrusion process are shown in Supplementary Fig. 9. The barrier for the minor groove extrusion is 17.9 kcal/mol. This result can be compared with the different results reported for MutM between the major^30,42^ and minor groove base extrusions^22,29^.

In summary, we present X-ray crystallographic structures of human DNA glycosylase hOGG1 interrogating DNA lesions in their intrahelical position, achieved by covalent trapping of an ordinarily transient state in DNA recognition. They reveal how hOGG1 discriminates oxoG from G while both are embedded in the DNA duplex. Specifically, the enzyme utilizes unique protein/DNA contacts to induce DNA bending at the target site. This bending brings the repulsive functional group of oxoG to the immediate vicinity of the DNA backbone, resulting in an oxoG specific distortion of the DNA backbone in its intrahelical orientation. In silico molecular dynamics simulations and free energy calculations corroborate the structural results and help to elucidate the role of the human enzyme in discriminating oxoG from G prior to a complete extrusion from the DNA stack. The results presented here broaden our understanding of one of the earliest events that occur as this extraordinary enzyme patrols genome in its surveillance of DNA damage.

## Methods

### Cross-linked complex formation and crystallization

A fragment of hOGG1 (amino acids 12–327, UniProtKB-015527) bearing the Y207C, Y207C/C253W, and Y207C/K249Q mutation was expressed in *Escherichia coli* BL21(DE3)pLysS cells. The cells were lysed by sonication in solution of 50 mM sodium phosphate pH 8.0, 10 mM imidazole, 500 mM NaCl, 5 mM BME, and 10% glycerol. The protein was immobilized by Ni-NTA resin (Qiagen) and eluted with 50 mM sodium phosphate pH 8.0, 250 mM imidazole, 500 mM NaCl, 5 mM BME, and 10% glycerol. Protein was concentrated, centrifuged, and diluted with 10 mM Tris pH 7.4 to 50 mM NaCl, loaded to Hi-Trap SP column (GE Healthcare) and eluted with increasing NaCl concentration. The N-terminal histidine-tag was cleaved by enterokinase digestion (New England Biolabs) using a 1:1 solution of 1 M CaCl_2_ for 36 h at 4 °C. Protein was further purified by Superdex-200 gel filtration chromatography (GE Healthcare) equilibrated with 10 mM Tris 7.4, 100 mM NaCl, 1 mM EDTA, and 10% glycerol^43^. Each mutant was prepared using QuickChange mutagenesis kit (Stratagene) and confirmed by sequencing (see Supplementary Table 2 for primer sequences used in mutagenesis).

Phosphoramidite derivatives of 8-oxoG and 2-F-dI were purchased from Glen research. DNA oligomers 5′-AGCGTCCAXG*TCTACC-3′, where X denotes 8-oxoG or G and G* refers the site of modification with the thiol-bearing tether, were synthesized using ABI Expedite 8909 DNA synthesizer and functionalized with X_8_ (NH_2_CH_2_CH_2_OCH_2_CH_2_OCH_2_CH_2_S–)_2_ using post synthetic modification^44^. DNA oligonucleotides were deprotected with ammonium hydroxide and purified in 20% denaturing urea polyacrylamide gel electrophoresis (PAGE). For DNA containing tether and oxoG on the same strand, 50 μM β-me was added to prevent oxidative degradation of 8-oxoG. DNA was purified by 20% urea-PAGE and dissolved in 10 mM Tris, pH 8.0, 1 mM EDTA, and annealed with complimentary strand 5′-TGGTAGACCTGGACGC-3′.

Cross-linked complexes were formed by mixing duplex DNA with 2-fold molar excess protein and incubating at 4 °C for several days. Unreacted DNA and protein were removed by Mono Q chromatography (GE Healthcare). The purified complexes were buffer-exchanged to 10 mM Tris 7.4 and 100 mM NaCl, concentrated and crystallized by hanging droplet vapor diffusion at 20 °C. For each complex, crystals were allowed to grow for several days, transferred to a cryoprotectant solution containing mother liquor supplemented with 25% glycerol, and frozen in liquid nitrogen for data collection. For LRC (Y207C/K249Q), diffraction quality crystals appeared with 16.4 mg/mL complex concentration (protein concentration measured using Bradford assay) within a few days in well solution containing 100 mM sodium cacodylate, pH 6.5, 200 mM MgCl_2_ and 18% polyethylene glycol 8000. For IC (Y207C), diffraction quality crystals appeared with 12 mg/mL complex concentration within a few days in well solution containing 200 mM NH_4_NO_3_, and 20% polyethylene glycol 3350. For EC (Y207C/C253W), diffraction quality crystals appeared with ~17 mg/mL complex concentration within a few days in well solution containing 100 mM sodium cacodylate, pH 6.1, 200 mM MgOAc, and 17% polyethylene glycol 8000.

### Structure determination

Diffraction datasets were collected at −170 °C at the 24-ID-C and 24-ID-E beamlines (NE-CAT) of the Advanced Photon Source and processed using the HKL program suites^45^. Initial molecular replacement solutions were obtained by PHASER in the CCP4 suite^46,47^, using the coordinates of previously determined hOGG1 structure (PDB ID: 1EBM^16^) but omitting DNA as search models. Each hOGG1–DNA model was built through iterative cycles of manual model building in COOT^48^ and structure refinement using REFMAC5^49,50^ and PHENIX^51^. The Ramachandran plots, calculated by MolProbity (http://molprobity.biochem.duke.edu), confirmed no residues in disallowed regions for all structures. Full details on the data collection and structure refinement are provided in Supplementary Table 1. PyMol (The PyMOL Molecular Graphics System, Version 2.0, Schrödinger, LLC.) was used to prepare all structure model figures presented in the paper.

### System preparation for molecular dynamics (MD) simulations

Two systems were prepared based on the intrahelical IC (for G) and EC (for oxoG) structures, respectively. In the preparation of the IC system, we further refined the IC X-ray structure to build more base pairs. Although this resulted in slightly lower quality of DNA structure, the core DNA base pairs were essentially unchanged. We used this refined structure in the IC system building. In each system, protonation states of all ionizable residues were determined based on their hydrogen bonding interactions deduced from the X-ray structures as well as on their pKa values in water. All crystal waters were included. For DNA, the central 14 base pairs of the sequence presented in Fig. 1d were used in the simulations, in which any missing nucleotide coordinates from the crystal structures were model built as the standard B-form DNA. Then, the HBUILD facility of the CHARMM program^52,53^ was used to assign  atomic coordinates of hydrogen atoms. The resulting systems were solvated with a rhombic dodecahedron (RHDO) box of 11,712 TIP3P water molecules^54^ and any water molecules^42^ within 2.5 Å from any heavy atom of protein, DNA, and crystal water were removed, leaving, for example, 9554 TIP3P waters for the IC system. Finally, each system was neutralized by adding 50 Na^+^ and 26 Cl^−^ ions at random positions, making its ionic concentration equal to 150 mM. In addition, two additional systems were prepared based on the LRC X-ray structure (PDB-ID: 1YQR)^23^ to be used as the reference state of the targeted molecular dynamics (TMD) simulations^55^ (see the Supplementary Methods for details).

### MD simulations

Each system was first energy minimized for 5000 steps and equilibrated for 500 ps at 300 K. The energy minimization and equilibration procedures were very similar to those employed in our previous studies of MutM/DNA complexes^22,29^. Then, the production MD was carried out for 500 ns for the IC and EC systems, during which the atomic coordinates of the entire system were saved at every 2 ps for later analysis. The all-atom CHARMM22^56^ and 27 force fields^57^ were used to represent the protein, DNA, and ions, the CMAP correction^58^ for protein backbone dihedrals, and the TIP3P water model^54^ for water molecules, respectively. For oxoG, we used the force field parameters developed in our previous study^22^. The RHDO periodic boundary conditions were imposed with the lattice length parameter of 78.5 Å. Electrostatic interactions were evaluated using the smooth particle mesh Ewald (PME) sum method^59^ and van der Waals interactions were evaluated using a switching function between 9.0 Å and 11.0 Å. All MD simulations were performed with a 2 fs integration time step and SHAKE^60^ applied to all bonds involving hydrogen atoms. The Langevin thermostat was used to maintain the system temperature at 300 K. In all simulations, we also applied harmonic restraints to the terminal base pairs to avoid them fraying away from each other.

System preparations, trajectory analysis, the TMD simulations, and the string method simulations^36^ (see below) were carried out using the CHARMM program (version c37a1)^52,53^ and the 500 ns production MD simulations were performed using the NAMD program^60^.

### String method (SM) simulations

The base extrusion pathways for G and oxoG were determined by applying the string method in collective variables (SMCV)^36^. In SMCV, a path connecting two end state conformations (i.e., the intrahelical IC/EC and LRC conformations) is represented by *N* discretized images (called MD replicas), which are evenly distributed along the path. In the present work, we used *N* = 64 discretized images to represent the entire base extrusion pathway described by a total of 45 CVs defined in Supplementary Fig. 10a. Starting from the initial path generated by the TMD simulation (Supplementary Methods), each path was optimized for 25 ns in an iterative manner. A total of 1.28 μs path optimization MD was performed. Then, the Markovian milestoning simulations with Voronoi tessellations^36,61^ were performed for 10 ns for each MD replica (thus, 0.64 μs MD simulations collectively) to determine the free energy change along the optimized base extrusion paths. The details of the SMCV path optimization and Voronoi tessellations simulations are provided in the Supplementary Methods.

### Reporting summary

Further information on research design is available in the Nature Research Reporting Summary linked to this article.

## Supplementary information

Supplementary Information

Descriptions of Additional Supplementary Files

Supplementary Movie 1

Reporting Summary

## Data Availability

Atomic coordinates and structure factors of the lesion recognition, interrogation, and encounter complexes have been deposited in the Protein Data Bank with accession code, 6W0M, 6W0R, 6W13, respectively. Other data are available from the corresponding authors upon reasonable request.
