# Supplementary material for: The trajectory of intrahelical lesion recognition and extrusion by the human 8-oxoguanine DNA glycosylase
Source: Nat Commun. 2020 Sep 7;11:4437. doi: 10.1038/s41467-020-18290-2 (PMC7477556; doi:10.1038/s41467-020-18290-2)
Supplement: Supplementary file 1 — Supplementary Information [file 41467_2020_18290_MOESM1_ESM.pdf]

Supplementary Information

The Trajectory of Intrahelical Lesion Recognition and Extrusion by the Human 8-oxoguanine DNA Glycosylase

Uddhav K. Shigdel et al.

Supplementary Methods

Synthesis of Compound 2.

Compound 1 (Supplementary Figure 1) was dissolved in DMF and 1.1 equivalent of potassium phthalimide along with 1.1 potassium iodide was added to the reaction mixture. The mixture was allowed to react at 110 °C for at least 24 hrs. Then, the mixture was diluted with water, extracted with CH_2Cl_2 and dried over $MgSO_4$. The crude product was subjected to silica gel chromatography (ethylacetate: hexane solvent system) that yielded pure compound 2 (Supplementary Figure 1). ^{1}H NMR (500 MHz, $CDCl_3$) δ 2.55 (s, OH, 1H), 3.48-3.55 (t, 2H), 3.56-3.68 (m, 6H), 3.70-3.78 (t, 2H), 3.85-3.93 (t, 2H), 7.66-7.74 (m, 2H), 7.80-7.88 (m, 2H). MS (ESI) m/z, calculated $[M + H]^+$, 280.11, found 280.11.

Synthesis of Compound 3.

Carbon tetrabromide (2 eq) and triphenylphosphine (2 eq) were dissolved in THF at 0 °C and compound 2 was added dropwise. The reaction mixture was allowed to come to room temperature and stirred further for 2-3 hours. The reaction was quenched with water and the product was extracted with CH_2Cl_2 and dried over $MgSO_4$. Silica gel chromatography afforded pure compound 3 (Supplementary Figure 1). ^{1}H NMR (500 MHz, $CDCl_3$) δ 3.52-3.70 (m, 6H), 3.71-3.80 (m, 4H), 3.81-3.90 (t, 2H), 7.69-7.71 (m, 2H), 7.81-7.84 (m, 2H). MS (ESI) m/z, calculated $[M + H]^+$, 342.03, found 342.03 : 344.03 (1:1).

Synthesis of Compound 4.

Compound 3 was dissolved in 1:1 mixture of water: methanol, 1.2 equivalents of sodium thiosulfate were added, and the reaction mixture was refluxed. TLC was used to monitor the disappearance of compound 3. After the completion of the reaction, the reaction mixture was

cooled, and iodine (same equivalent as sodium thiosulfate) was added and refluxed again. After the removal of methanol, CH_2Cl_2 was added to the reaction mixture and washed with saturated sodium thiosulfate to remove excess I_2. After extraction and drying over $MgSO_4$, silica gel chromatography yielded pure compound 4 (Supplementary Figure 1). ^1H NMR (500 MHz, $CDCl_3$) δ 3.35-3.40 (t, 4H), 3.55-3.60 (m, 8H), 3.60-3.75 (m, 8H), 3.76-3.90 (t, 4H), 7.65-7.67 (m, 4H), 7.77-7.80 (m, 4H).

Synthesis of Compound 5.

Compound 4 was dissolved in 40% methylamine and was refluxed overnight under argon. The reaction was quenched with water and the final product was extracted at least 10 times with chloroform: methanol (3:1) yielding final product X8 (Supplementary Figure 1). ^1H NMR (500 MHz, $CDCl_3$) δ 2.84-2.88 (m, 8H), 3.48-3.50 (t, 4H), 3.58-3.63 (m, 8H, NH_2, 4H), 3.70-3.72 (t, 4H). MS (ESI) m/z, calculated $[M + H]^+$, 329.15, found 329.15.

Targeted Molecular Dynamics (TMD) Simulations for the Determination of the Initial Base Extrusion Path.

To yield an initial base extrusion pathway, TMD simulations[2] were performed between the intrahelical IC/EC structure and the LRC structure. Each system (i.e., the G and oxoG systems) was first equilibrated for 25 ns, followed by a total of 12 ns TMD simulations. During the TMD simulation, the root-mean-squared distance (RMSD) of the protein/DNA complex relative to the target structure (i.e., the LRC conformation) was holonomically decreased by 0.5×10^{-6} Å at each MD step. The atoms to which the TMD perturbations were applied are presented in Supplementary Figure 11. The simulations yielded the base extrusion along the major groove of DNA. For the system with oxoG, we also prepared the minor groove base extrusion pathway. Thus determined base extrusion pathways were then used as the initial paths of the string method (SM) simulations.

SMCV Path Optimization.

The path optimization was performed in an iterative manner. In each iteration, a short MD (0.1 ps during the first 10 ns with 32 MD replicas and 1 ps during 15 ns with 64 replicas) was first performed, during which each MD replica was harmonically restrained with a force constant of 100.0 kcal/mol-Å^2 to each CV value describing the path and local free energy gradient associated with each CV was evaluated. Then, the path was updated by evolving the path, i.e., the CV positions of the discretized images, in the direction opposite to that of the local free energy gradient. Finally, the CV positions of each image was reparametrized to enforce an equal arc-length distance between neighboring images[1], which completes one path optimization iteration cycle.

Milestoning Simulations with Voronoi Tessellations.

First, we partitioned the entire base extrusion pathway into N=64 Voronoi cells, each centered around each image position on the path. This partitioning defines the cell boundaries with neighboring cells, providing a local approximation of the hyperplanes defining the isocommittor surfaces along the base extrusion pathway. Then, from each Voronoi cell, MD simulations were performed for 10 ns (thus, 0.64 μs MD simulations collectively), while confining each MD replica to remain inside each cell. This was achieved by imposing a reflection rule at cell boundaries, i.e., all particle momenta were reversed when the simulation reached the cell boundary. During the simulation, the number of collisions that each MD trajectory made with each cell boundary was counted. Finally, the collisions counted were used to estimate the equilibrium probability to find the system in each cell and its associated free energy.

Supplementary Table 1. Data collection and refinement statistics

	xLRC	IC	EC
**Data collection**			
Space group	$P6_522$	$P6_522$	$P6_522$
Cell dimensions			
a, *b*, *c* (Å)	91.68, 91.68, 212.11	88.93, 88.93, 210.54	90.71, 90.71, 210.80
α, β, γ (°)	90.0, 90.0, 120.0	90.0, 90.0, 120.0	90.0, 90.0, 120.0
R_{merge}	0.086 (0.746)	0.17 (0.989)	0.124 (1.250)
$I/\sigma I$	35.2 (5.9)	10.3 (2.0)	17.0 (2.4)
Completeness (%)	99.83 (98.36)	99.92 (99.87)	99.92 (99.61)
Redundancy	20.7 (21.2)	5.8 (5.8)	10.5 (10.8)
Unique reflections	22361	21524	21460
Resolution (Å)	45.84-2.37 (2.43-2.37)	43.50-2.35 (2.45-2.35)	45.35-2.38 (2.44-2.38)
**Refinement**			
$R_{work/}$ R_{free}	0.197/0.265	0.208/0.249	0.214/0.271
No. atoms			
Protein	2495	2465	2490
Ligand/ion	11	22	28
Water	100	115	85
DNA	575	224	244
B-Factors (Å^2)			
Protein	42.2	35.1	49.6
DNA	87.7	92.2	108.9
Water	43.5	33.9	47.2
Ligand/Ions	69.9	54.8	78.8
R.m.s deviations			
Bond lengths (Å)	0.009	0.004	0.008
Bond angles (º)	1.448	0.770	1.361

[a]Values in parentheses are for the last resolution shell

Supplementary Table 2: Primer sequences used in mutagenesis

Mutant	Primer	DNA sequence
Y207C	Forward	5'-GCTATCGTGCCCGTTGCGTGAGTGCCAGT-3’
	Reverse	5'-ACTGGCACTCACGCAACGGGCACGATAGC-3'
K249Q	Forward	5’-TGGAGTGGGCACCCAGGTGGCTGACTG-3'
	Reverse	5’-CAGTCAGCCACCTGGGTGCCCACTCCA-3'
C253W	Forward	5’-AAGGTGGCTGACTGGATCTGCCTGATGGC-3'
	Reverse	5’-GCCATCAGGCAGATCCAGTCAGCCACCTT-3'

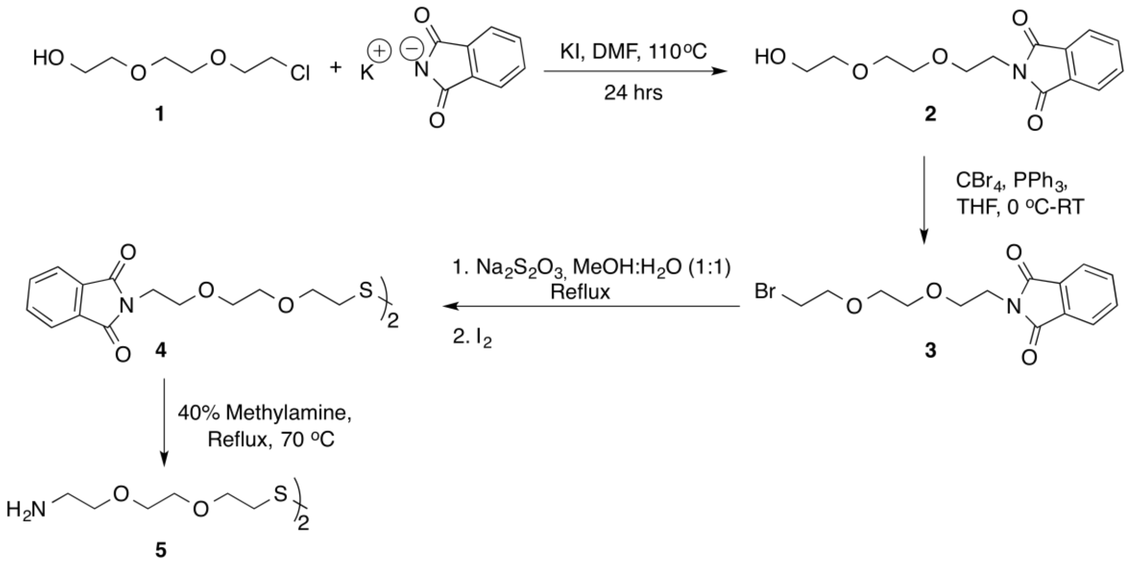

Supplementary Figure 1. Synthetic scheme of X8 linker.

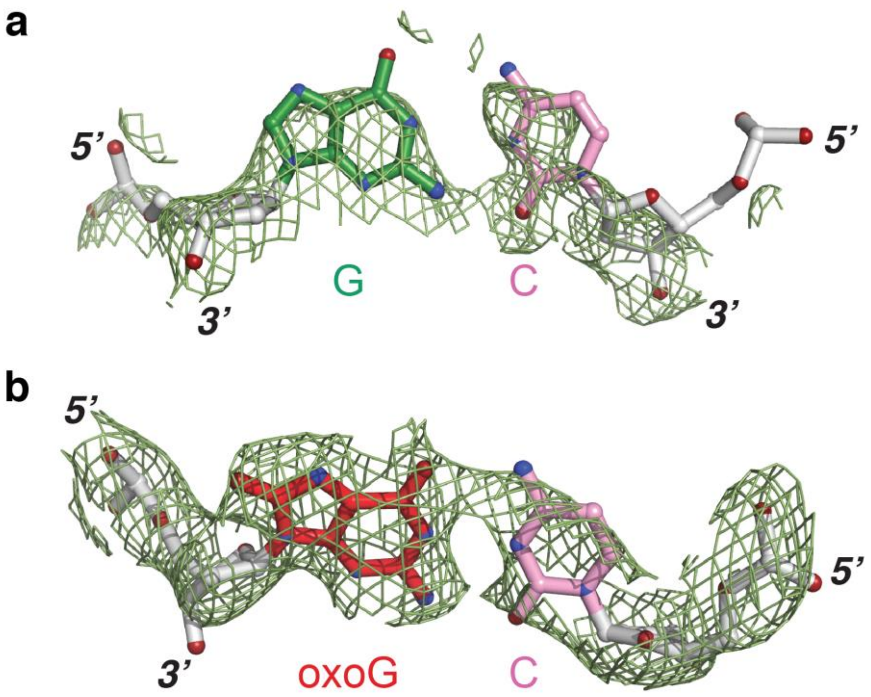

Supplementary Figure 2. ***F*o-*F*c omit electron density maps at 2.5 σ showing intrahelical state for (a) target G:C and (b) oxoG:C base pair.** The target bases, G and oxoG are colored green and red, respectively.

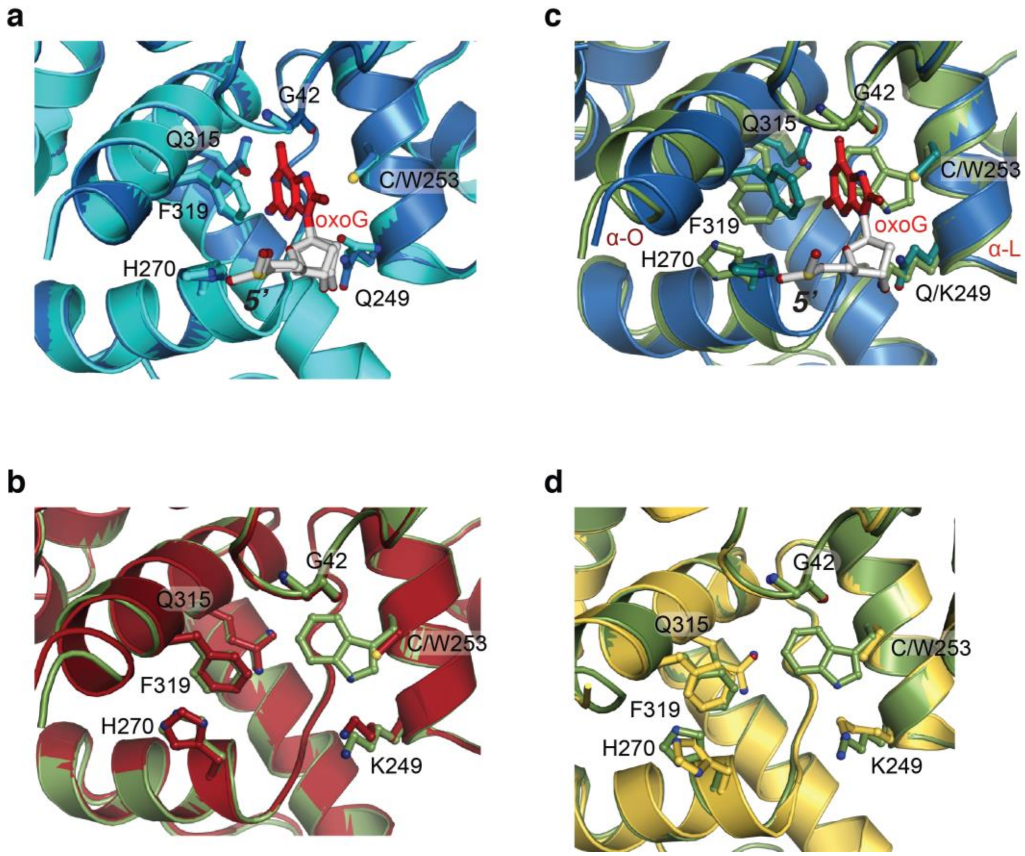

Supplementary Figure 3. Close-up views of the active-site of hOGG1. Color-coding is as in Figs. 2 and 4. Key residues in oxoG recognition, H270, F319, Q315, G42, C253, K249, and oxoG base are shown in sticks. **a,** Active-site comparison of xLRC and native LRC (PDB: 1EBM, cyan); **b,** active-site comparison of EC and IC structures; **c,** active-site comparison of EC and xLRC structures; **d,** active-site comparison of EC and Exo-site (PDB, 1YQK, yellow orange) structures.

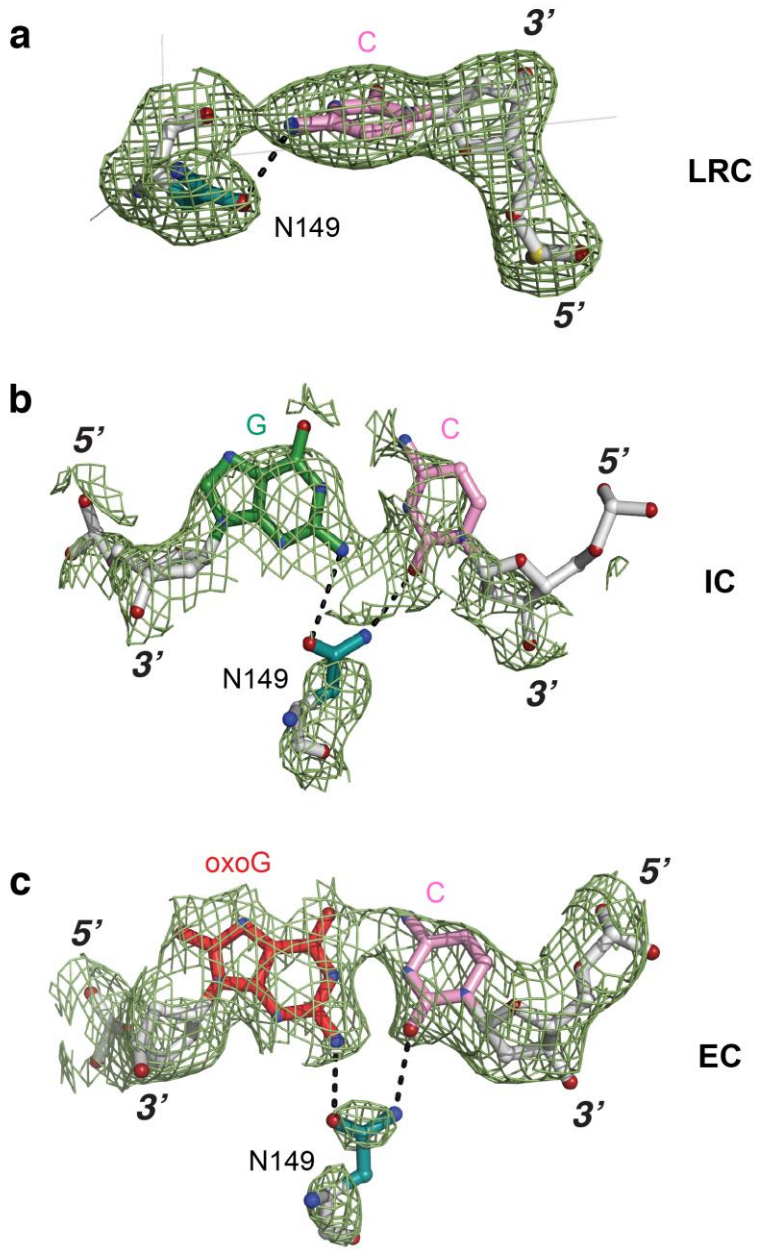

Supplementary Figure 4. F_o-F_c omit electron density maps at 2.5 σ showing N149 interaction with (a) C of xLRC (b) target G:C and (c) oxoG:C base pair. The target bases, G and oxoG are colored green and red respectively, C pink and N149 deep teal.

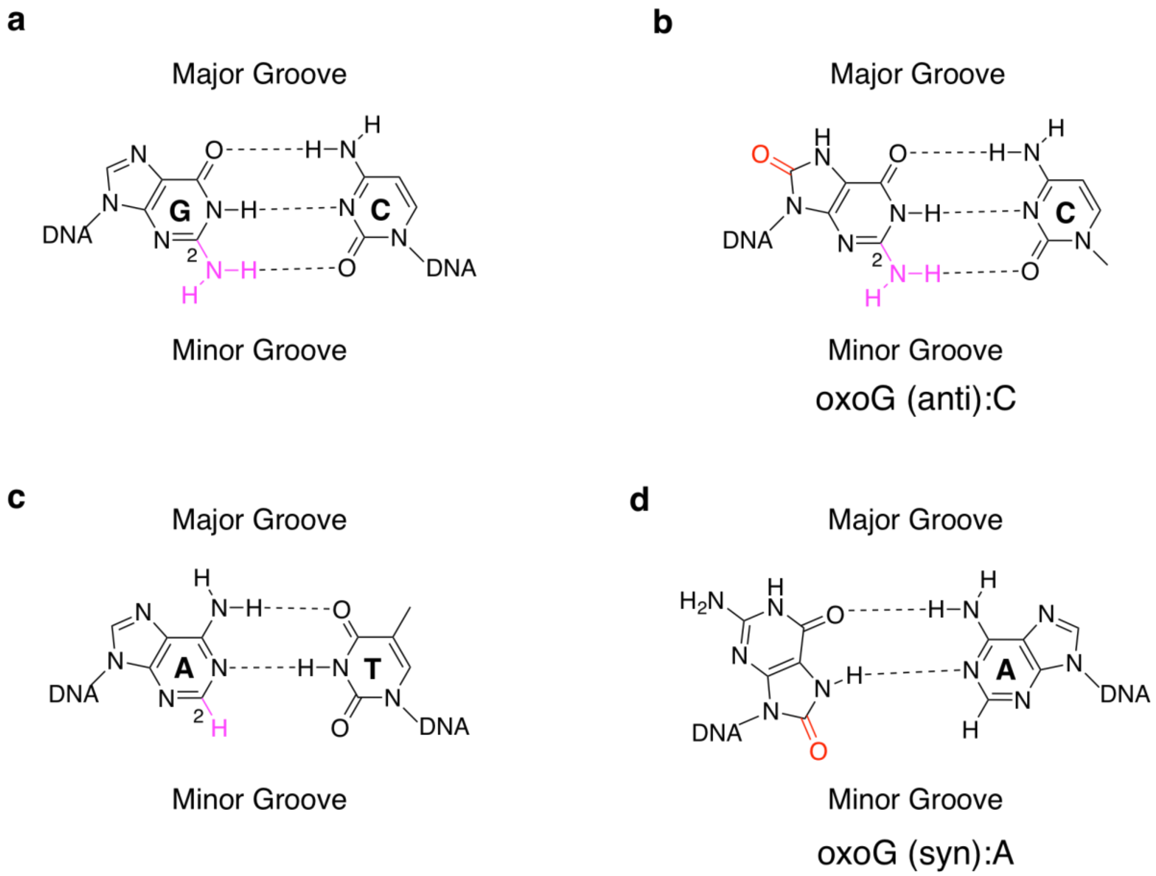

Supplementary Figure 5. Major groove and minor groove face of DNA base-pair (a) G:C, (b) oxoG (anti):C and (c) A:T and (d) oxoG(syn):C. The major difference in the minor groove face of G:C and A:T base pairs is at the 2 position of purine base G and A. Whereas G at 2 position can participate in hydrogen bonding interaction with N149, because A at 2 position cannot donate hydrogen, it is devoid of hydrogen bonding interaction with Asn149. oxoG (syn):A could form non-productive interaction.

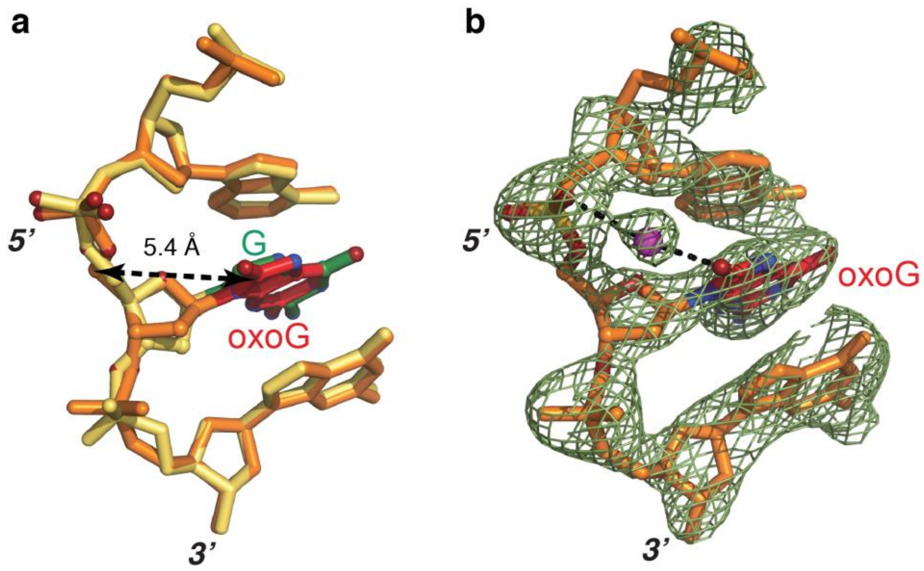

Supplementary Figure 6. Comparison of target nucleobase G and oxoG. Only the target strand is shown for simplicity. DNA bases are shown as orange sticks in EC, and as yellow orange in IC. The target base, oxoG and G are colored red and green respectively. **a** Overlay of target-strand of EC and IC structures. The distance between C8 and C5’ of oxoG, shown as dashed arrow is 5.4 Å, whereas for G is 4.4 Å. **b** Target-strand of EC showing F_o-F_c map, 2.5 σ, for water molecule which is colored magenta. The water molecule interacts with the carbonyl of oxoG and a non-bridging oxygen on the 5’ of oxoG. This water molecule is absent in the IC structure.

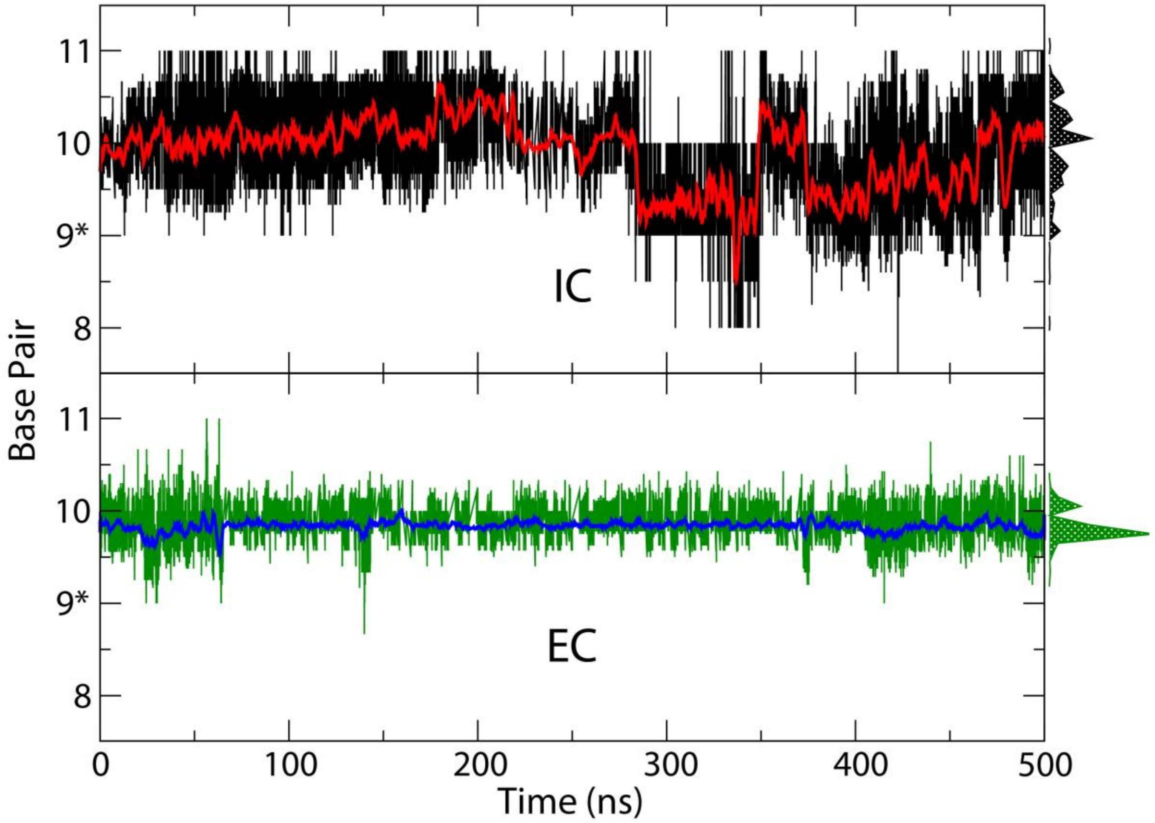

Supplementary Figure 7. Time traces of mean contact positions of hOGG1 along the target DNA strand. The mean position (N) of the protein along the DNA strand is defined as $N = (\sum_i n_i R_i)/(\sum_i n_i)$, where n is the number of protein residues within 4.5 Å from the i-th nucleotide phosphorus atom and R is the nucleotide number (Fig. 1). 9* indicates the position of the target nucleotide, i.e., G for IC (upper panel) and oxoG for EC (lower panel). The thick lines in each figure represent the running averages over 1 ns around each time point, and on the right side of each figure, the density distribution of the contact position is shown.

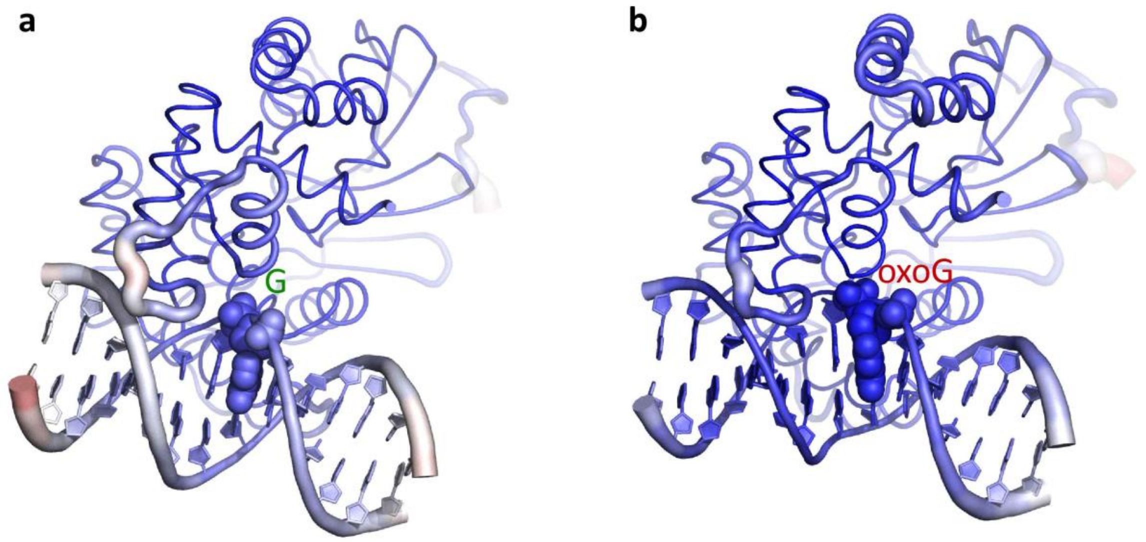

Supplementary Figure 8. Root-mean-square fluctuation (RMSF) of each atom around the average structure determined from the 500 ns MD simulation: (a) IC system and (b) EC system. In each structure, the RMSF values are represented by the thickness of the tube and color coded from blue to white then to red with the increase of the RMSF value.

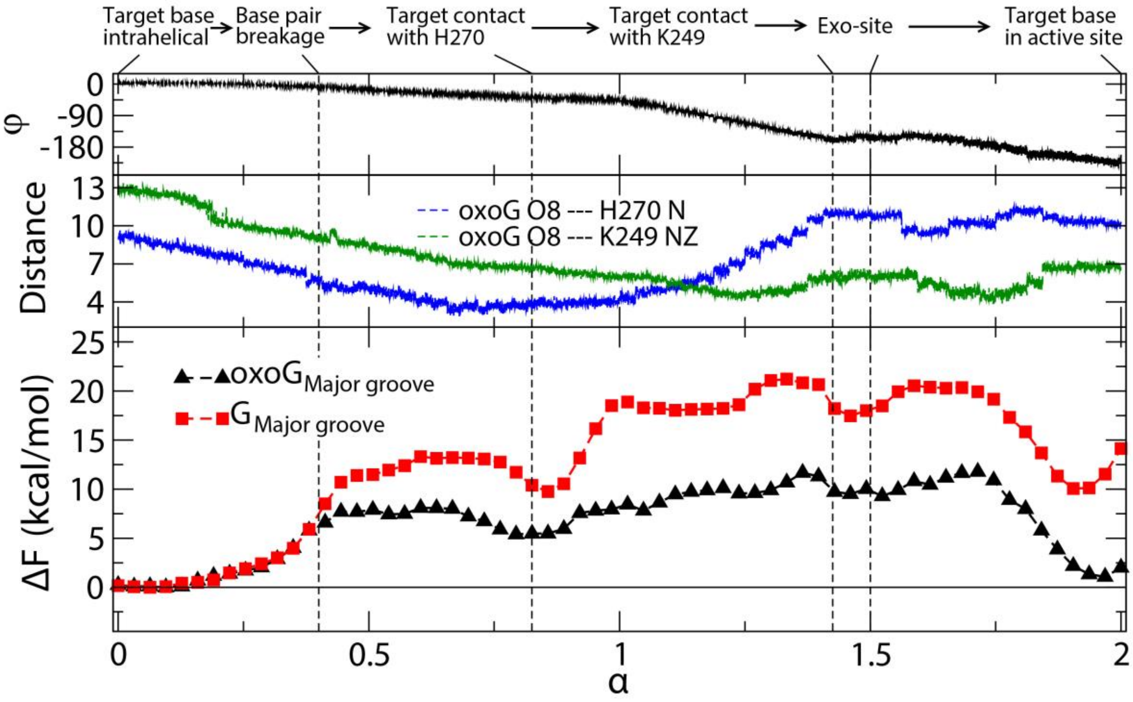

Supplementary Figure 9. Free energy (FE) profiles of the entire base extrusion process along the major groove pathway for hOGG1. The entire pathway is described by a progress variable α, which varies between 0.0 (fully intrahelical state) and 2.0 (the active site bound state). In Fig. 5a, we present the FE profile between α = 0.0 and 1.0, which corresponds to event (1) described in the main text. Event (2), i.e. the exo-site binding, corresponds to the region 1.0 < α < 1.55, and finally event (3), the active site binding corresponds to the region α > 1.55, respectively. Important events along the base extrusion process are indicated in the figure. In the upper panel of the figure, we present the change of pseudo-dihedral angle (defined in Supplementary Figure 10b) along the base extrusion process for oxoG and in the middle panel, the distances (Å) of oxoG C8=O to H270 backbone N and also to K249 NZ.

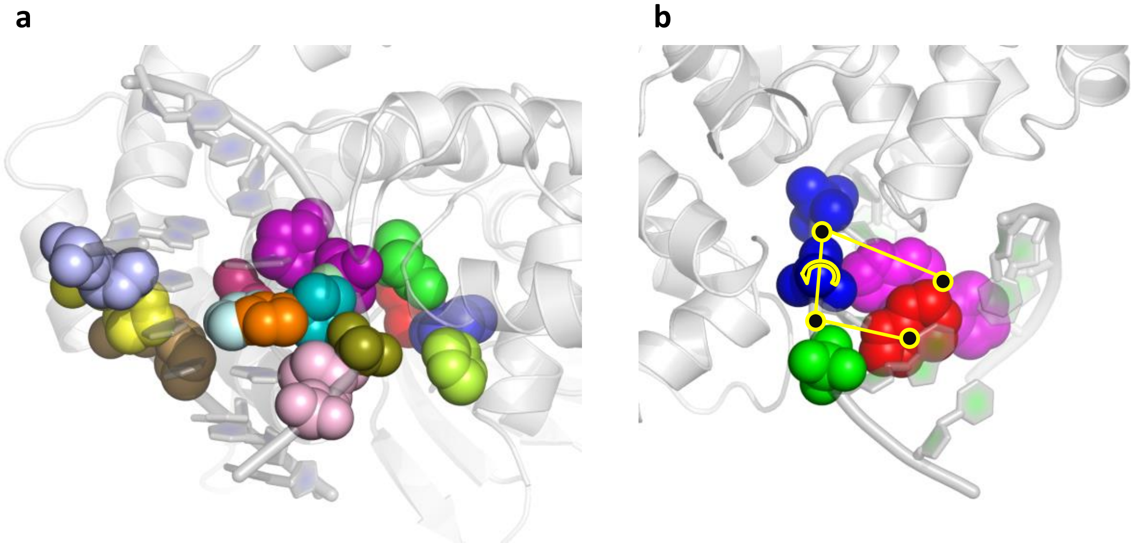

Supplementary Figure 10. Definitions of (a) collective variables (CVs) used in the string method (SM) simulation and (b) pseudo-rotation angles describing the progress of base extrusion. a Total of 15 points are defined, each of which is defined as the center of mass of each collection of atoms represented by van der Waals sphere and different colors, and the collective variables are the Cartesian coordinates of each point, thus a total of 45 CVs. **b** The center of mass of each collection of atoms represented by van der Waals sphere and different colors are used to define the pseudo-rotation angle, describing the extend of base extrusion out of the DNA helix. The phosphate group in the middle (one of the blue phosphate groups close to the green phosphate) is also used to define the 2nd center of mass position together with the green phosphate. Therefore, the 2nd dot is located in the middle between the green and the blue phosphates. The third dot is located between the two blue phosphate groups.

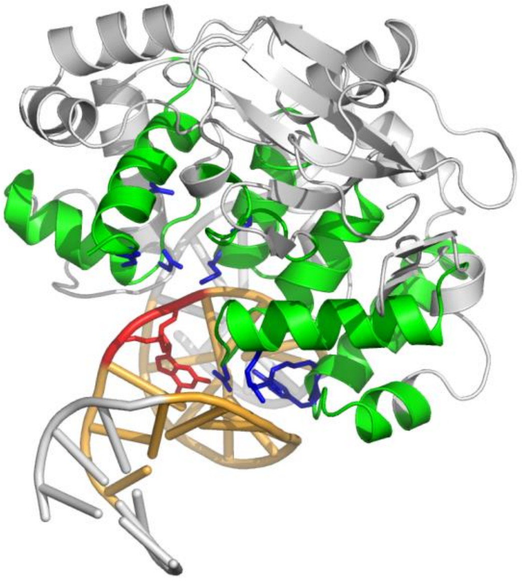

Supplementary Figure 11. Color-coded cartoon of hOOG1 with G-containing DNA in its intrahelical conformation (i.e., IC). The atoms, to which the TMD perturbation was applied, were presented with green for protein residue backbones, blue-stick for protein residues with side chains, gold for DNA residues and finally red-stick for the target G, respectively. The rest of the complex was shown in light gray. The same set of atoms were selected for the TMD simulations with oxoG.

Supplementary References

1. Brooks, B. R. *et al.* CHARMM: the biomolecular simulation program. *J Comput Chem* **30**, 1545–614 (2009).

2. Schlitter, J., Engels, M., Krüger, P., Jacoby, E. & Wollmer, A. Targeted Molecular Dynamics Simulation of Conformational Change-Application to the T ↔ R Transition in Insulin. *Mol Simulat* **10**, 291–308 (1993).
