# Supplementary material for: The trajectory of intrahelical lesion recognition and extrusion by the human 8-oxoguanine DNA glycosylase
Source: Nat Commun. 2020 Sep 7;11:4437. doi: 10.1038/s41467-020-18290-2 (PMC7477556; doi:10.1038/s41467-020-18290-2)
Supplement: Supplementary file 2 — Descriptions of Additional Supplementary Files [file 41467_2020_18290_MOESM2_ESM.pdf]

Descriptions of Additional Supplementary Files

Supplementary Movie 1

Description: String method (SM) simulations for extrusion of oxoG out of the DNA duplex, catalyzed by hOGG1. hOGG1 is shown in cyan cartoon, DNA in green stick, in which each phosphorus atom is shown in a sphere, and oxoG in van der Waals sphere. Several residues including G42, C253, K249, H270, Q315 and F319, are shown as sticks.
